# Risdiplam improves subjective swallowing quality in non-ambulatory adult patients with 5q-spinal muscular atrophy despite advanced motor impairment

**DOI:** 10.1007/s00415-024-12203-9

**Published:** 2024-02-15

**Authors:** S. Brakemeier, J. Lipka, M. Schlag, C. Kleinschnitz, T. Hagenacker

**Affiliations:** Department of Neurology and Center for Translational Neuro- and Behavioral Sciences (C-TNBS), University Hospital Essen, Hufelandstr. 55, 45147 Essen, Germany

**Keywords:** Spinal muscular atrophy, Risdiplam, ALSFRS-R, SSQ, Swallowing

## Abstract

**Background:**

5q-associated spinal muscular atrophy (SMA) is characterized by the progressive loss of motor neurons with consecutive weakness and atrophy of the limb, respiratory, and bulbar muscles. While trunk and limb motor function improve or stabilize in adults with SMA under nusinersen and risdiplam treatment, the efficacy on bulbar function in this age group of patients remains uncertain. However, it is important to assess bulbar dysfunction, which frequently occurs in the disease course and is associated with increased morbidity and mortality.

**Methods:**

Bulbar function was evaluated prospectively in 25 non-ambulatory adults with type 2 and 3 SMA before and 4 and 12 months after risdiplam treatment initiation using the *Sydney Swallow Questionnaire* (SSQ) and the bulbar subscore of the *Amyotrophic Lateral Sclerosis Functional Rating Scale Revised* (b-ALSFRS-R). Extremity function was assessed using the *Hammersmith Functional Motor Scale Expanded* (HFMSE) and *Revised Upper Limb Module* (RULM).

**Results:**

Subjective swallowing quality, measured with the SSQ, improved after 12 months of therapy with risdiplam. For the b-ALSFRS-R, a non-significant trend towards improvement was observed. The RULM score improved after 12 months of risdiplam therapy, but not the HFMSE score. HFMSE and RULM scores did not correlate with the SSQ but the b-ALSFRS-R score at baseline.

**Conclusions:**

The improvement in subjective swallowing quality under risdiplam treatment, despite an advanced disease stage with severe motor deficits, strengthens the importance of a standardized bulbar assessment in addition to established motor scores. This may reveal relevant treatment effects and help individualize treatment decisions in the future.

## Introduction

5q-associated spinal muscular atrophy (SMA) is a hereditary motor neuron disease that leads to progressive weakness and muscular atrophy including ventilatory and bulbopharyngeal muscles. SMA is caused by homozygous deletion or compound heterozygosity with deletion and point mutation in the survival motor neuron 1 (*SMN1*) gene [1]. The lack of functional SMN protein results in motoneuronal degeneration in the anterior horn of the spinal cord [2]. Truncated SMN protein deriving from the *SMN2* gene of variable copy number cannot sufficiently compensate for the absence of functional SMN protein [3]. As classified by the natural history of the disease, SMA phenotypes are differentiated according to the age at onset and the highest achieved motor milestones, with later-onset SMA types 2 and 3 being associated with a milder disease course and a higher *SMN2* copy number [4, 5]. However, all phenotypes share progressive loss of motor skills and achieved motor milestones during the natural disease course [6].

With evolving treatment options for SMA over the past few years, three gene-based treatment options are now available. For adult patients, two of these are presently in frequent clinical use: the antisense-oligonucleotide nusinersen and the small molecule risdiplam, both of which alter the splicing of *SMN2* pre-mRNA to resemble that of *SMN1*, producing more functional SMN protein [7, 8]. Pivotal and real-world studies have demonstrated relevant motor improvements in adult SMA patients under nusinersen [9–11] and in SMA types 2 and 3 patients aged 2–25 years under risdiplam [12]. Other than for nusinersen, data on the efficacy of risdiplam in larger real-world cohorts of adult patients over longer observation periods are pending. Most studies have defined motor function assessments such as the *Hammersmith Functional Motor Scale Expanded* (HFMSE), *Revised Upper Limb Module* (RULM), and *Motor Function Measure* (MFM32) as efficacy outcome measures that reflect trunk and extremity function while excluding bulbar and ventilatory function. However, bulbar dysfunction affects a large proportion of patients with SMA and poses a functional burden and a risk for respiratory complications and malnutrition, with an increased mortality risk [13]. Bulbar symptoms include chewing and swallowing difficulties, reduced mandibular range of motion and strength, and aberrant craniofacial morphology [14]. Bulbar dysfunction typically occurs in the early and advanced disease stages, mostly in SMA types 1 and 2 [13–15]. In a previous study, we evaluated bulbar function in adults with SMA under nusinersen and found no improvement but preservation over 14 months of therapy [16]. For risdiplam, pivotal studies have shown preservation of swallowing function in SMA infants with sustained oral feeding ability over 12 months, indicating a relevant effect of risdiplam on bulbar function in this patient group [17]. Its efficacy in adults with SMA remains unclear. Here, we investigated the course of bulbar function in adult patients with SMA under risdiplam using two questionnaire-based measures: the *Sydney Swallow Questionnaire* (SSQ) and the bulbar subscore of the *Amyotrophic Lateral Sclerosis Functional Rating Scale Revised* (b-ALSFRS-R).

## Materials and methods

### Study design and participants

This study was conducted in the Department of Neurology, University Hospital, Essen, Germany. Data were prospectively collected between September 2020 (including patients from the Risdiplam Compassionate Use Program) and October 2022. Of 29 patients with molecularly confirmed types 2 and 3 SMA who had received risdiplam for at least 4 months by October 2022, 25 patients with bulbar dysfunction documented by patient-reported outcome measures before the initiation of treatment with risdiplam, without a percutaneous enteroscopic gastrostoma, and without prior SMN-enhancing therapy (nusinersen, onasemnogene abeparvovec) were included in the analysis. All patients had a homozygous deletion of the *SMN1* gene and were non-ambulatory. One upward outlier in terms of ambulation status and RULM and HFMSE baseline scores were excluded from analyses to prevent distortion of the results. The 12-month follow-up data were available for the SSQ in 19 patients and for the b-ALSFRS-R in 17 patients. Risdiplam was administered orally at a dose of 5 mg daily according to the label [18].

### Assessment of bulbar function

We used bulbar function items 1–3 of the *Amyotrophic Lateral Sclerosis Functional Rating Scale Revised* (b-ALSFRS-R) and the *Sydney Swallow Questionnaire* (SSQ) to assess bulbar function in this study, as published previously for patients treated with nusinersen [16]. Data were collected during regular follow-up of patients shortly before treatment initiation (T0) and at 4 months (T1) and 12 months (T2) after treatment initiation. The ALSFRS-R and SSQ are both questionnaire-based and were conducted in their German version [19, 20].

The SSQ is a patient-reported outcome measure containing 17 questions designed to assess the subjective severity of oropharyngeal dysphagia in several patient populations [21]. The SSQ has been found to be reliable, valid, and sensitive in patients with neurogenic, structural, and age-related swallowing difficulties [19]. Sixteen items query subjective swallowing difficulties, such as bolus consistency and signs of penetration, aspiration, and regurgitation. The score primarily reflects the quality of swallowing as perceived by patients. One item that assesses the duration of food intake was not included in the calculation of the total score because it does not specifically reflect swallowing function but rather includes the motor function of the upper extremities and may vary depending on assistance with food intake. Answers to the other 16 items were provided on a visual analog scale ranging from 0 to 100. The total score was calculated from 16 questions as an arithmetic mean of 0–100, with higher scores indicating more severe bulbar impairment.

The ALSFRS-R is a widely used and validated rater‐administered scale to measure physical function and disease progression in patients with amyotrophic lateral sclerosis (ALS) [22]. It consists of 12 items in four domains, with one domain representing bulbar function with 3 items addressing speech, salivation, and swallowing. A score of 0–4 is assigned to each item, with 0 indicating severe impairment and 4 indicating normal function. A possible score of 0–12 is obtained for the b-ALSFRS-R, with lower scores indicating more severe bulbar impairment.

In addition to the bulbar scores, the HFMSE and RULM scores were assessed. Both are established and validated tools for evaluating motor function and disease progression in patients with SMA types 2 and 3 [23, 24]. The HFMSE includes 33 items testing motor function of the extremities, trunk, and head, scored from 0–2, resulting in a maximum score of 66. Higher scores indicate better motor function. The RULM assesses upper extremity function using 19 items. One item is scored 0 or 1, and the remaining 18 are scored 0–2 points resulting in a maximum score of 37. Again, a higher score indicates better motor function.

### Statistical analyses

**Fig. 1:**
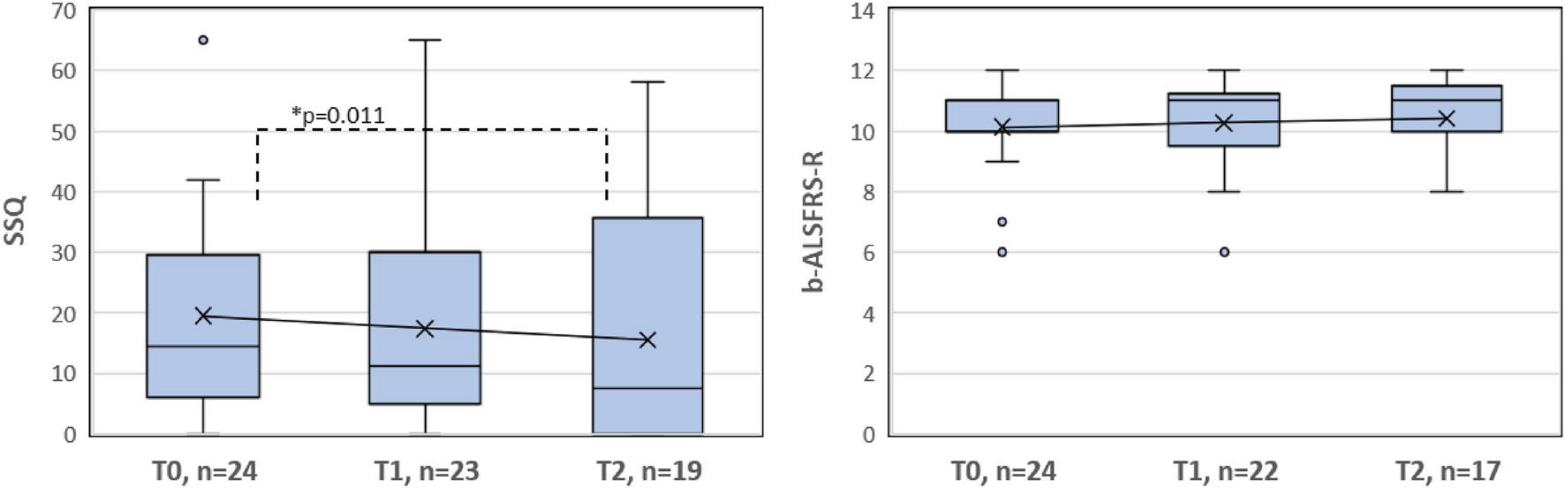
Measures of central tendency and dispersion and the mean SSQ and b-ALSFRS-R scores at T0, T1, and T2. The boxes are defined by the upper and lower quartiles. The median is marked as a continuous line in the box. The length of the whiskers is limited to a maximum of 1.5 times the interquartile range. Data points outside this range are marked as circles. Crosses represent the respective arithmetic means. Significant differences based on a significance level of *α* = 0.05 and calculated with the Wilcoxon signed-rank test are indicated by an asterisk *. Description: The mean SSQ values decrease from T0 to T1 and further to T2. The median values are lower than the mean scores for each time point. The difference in the SSQ between T0 and T2 is marked as significant. The mean b-ALSFRS-R values increase from T0 to T1 and further to T2. The median value is lower than the mean value at T0 and higher than the mean value at T1 and T2

A Wilcoxon signed-rank test was performed for pre–post comparisons of the b-ALSFRS-R, SSQ, HFMSE, and RULM scores between T0 and T1 and between T0 and T2. In two patients, only the SSQ or only the b-ALSFRS-R were available at baseline; in all others, both scores were available. HFMSE and RULM scores were complete at baseline. Missing follow-up data resulted from missed or postponed appointments, change of treatment center, or termination of therapy and were excluded in the respective pairwise comparison. Descriptive statistics summarizing our dataset were calculated using all available data at the respective time points. Additionally, correlations between the baseline values of the SSQ and b-ALSFRS-R and those of the HFMSE and RULM were calculated using Spearman’s rank correlation coefficient. In exploratory analyses, bulbar function scores were correlated with SMN copy number, age, and disease duration. The alpha value was set to ≤ 0.05. Figure 1 visualizes the course of the bulbar scores from T0 to T1 to T2 in boxplots with mean lines added.

## Results

Table 1 presents the demographic and clinical characteristics of the participants.

**Table 1:** Demographic and clinical characteristics of all patients (*n* = 25)

Sex	Male (%)	Female (%)
	11 (44)	14 (56)

SMA type	2 (%)	3 (%)
	24 (96)	1 (4)

*SMN2* copy number	2 (%)	3 (%)
	3 (12)	22 (88)

Clinical classification	Sitter (%)	Non-sitter (%)
	17 (68)	8 (32)

Spondylodesis	Yes (%)	No (%)
	14 (56)	11 (44)

Non-invasive ventilation	Yes (%)	No (%)
	10 (40)	15 (60)

	Mean (sd)	Minimum	Maximum
Age	34.32 (11.31)	19	58
Disease duration in years	32.08 (10.81)	17.6	56.9
Baseline HFMSE score	1.96 (2.32)	0	11
Baseline RULM score	8.84 (5.11)	0	16
Baseline Body Mass Index	19.70 (6.0)	8.9	32.9
Vital capacity in litres	0.98 (0.64)	0.25	2.76

sd = standard deviation

The SSQ score improved significantly from T0 to T2 (*z* = − 2.548, *p* = 0.011, *n* = 18), with 15 patients (83%) reporting improvement and 3 (17%) reporting a decline within this period. There was no significant change in SSQ scores from T0 to T1 (*z* = − 1.625, *p* = 0.104, *n* = 22). 14 (64%) patients reported improvement, 6 (27%) decline, and 2 (9%) no change in the SSQ score from T0 to T1. For the b-ALSFRS-R, the pre–post comparisons revealed no significant change from T0 to T1 (*z* = − 0.775, *p* = 0.438, *n* = 22) or from T0 to T2 (*z* = − 1.814, *p* = 0.07, *n* = 17). From T0 to T1, 9 (41%) patients reported improvement, and 4 (18%) decline while in 9 (41%) patients, the b-ALSFRS-R score remained unchanged from T0 to T1. From T0 to T2, the b-ALSFRS-R improved in 8 (47%) patients, worsened in 3 (18%) patients, and remained unchanged in 6 (35%) patients. Analysis of motor scores revealed a significant improvement in the RULM Score from T0 to T2 (*z* = 2.359, *p* = 0.018, *n* = 19). No significant improvement was found in the RULM Score from T0 to T1, or in the HFMSE Score from T0 to T1 and T0 to T2. The measures of the central tendency and dispersion of the SSQ, b-ALSFRS-R and motor Scores HFMSE and RULM at T0, T1, and T2 are presented in Table 2. Data distribution and results of the paired comparisons are shown in Fig. 1.

**Table 2:** Descriptive statistics for the b-ALSFRS-R, SSQ and motor scores HFMSE and RULM at T0 (baseline), T1 (4 months), and T2 (12 months)

	*n*	Mean (sd)	Minimum	Maximum	Lower quartile	Median	Upper quartile
b-ALSFRS-R (0–12)							
T0	24	10.13 (1.33)	6	12	10	10	11
T1	22	10.27 (1.67)	6	12	9.5	11	11.3
T2	17	10.41 (1.54)	6	12	10	11	11.5
SSQ (0–100)							
T0	24	19.52 (16.43)	0	65	6.0	14.4	29.6
T1	23	17.41 (16.93)	0	65	4.9	11.3	30.0
T2	19	15.55 (18.59)	0	58.1	0.0	7.5	35.6
HFMSE (0–66)							
T0	25	1.96 (2.32)	0	11	0.5	1	2.5
T1	23	2.13 (2.12)	0	8	0	2	3
T2	19	2.26 (2.35)	0	7	0	2	4
RULM (0–37)							
T0	25	8.84 (5.11)	0	16	5.5	9	13
T1	23	9.30 (5.28)	0	15	7	10	14
T2	19	9.16 (5.73)	0	16	6	10	15

sd = standard deviation

Correlation analyses between baseline bulbar function measurements and baseline motor scores showed a significant positive correlation between the b-ALSFRS-R and the HFMSE (*r* = 0.444, *p* = 0.030, *n* = 24) and RULM (*r* = 0.525, *p* = 0.008, *n* = 24). There were no significant correlations between the SSQ and the extremity scores HFMSE (*r* = − 0.138, *p* = 0.521, *n* = 24) or RULM (*r* = − 0.104, *p* = 0.628, *n* = 24). The two bulbar scores were negatively correlated (*r* = − 0.558, *p* = 0.006, *n* = 23). Correlational analyses did not reveal associations between the bulbar scores and age, *SMN2* copy number, or disease duration, whereas the HFMSE and RULM baseline scores were negatively correlated with age and disease duration. The results of the correlation analyses are presented in Table 3.

**Table 3:** Spearman correlations for baseline bulbar and extremity motor scores, SMN2 copy number, age, and disease duration

	Age	*SMN2* copy number	Disease duration in years	Baseline b-ALSFRS-R	Baseline SSQ	Baseline HFMSE
Baseline b-ALSFRS-R	− 0.248 (0.243)	− 0.197 (0.355)	− 0.272 (0.198)			
Baseline SSQ	− 0.092 (0.670)	0.282 (0.182)	− 0.011 (0.961)	*− 0.558 (0.006)		
Baseline HFMSE	*− 0.597 (0.002)	0.272 (0.189)	*− **0.592 (0.002)**	*0.444 (0.030)	− 0.138 (0.521)	
Baseline RULM	*− 0.453 (0.023)	− 0.077 (0.714)	*− **0.474 (0.019)**	*0.525 (0.008)	− 0.104 (0.628)	*0.678 (< 0.001)

Two-tailed significance is given in parentheses. Significant correlations based on a significance level of *α* = 0.05 and calculated using Spearman’s rank correlation coefficient are indicated by an asterisk *

## Discussion

Subjective swallowing quality, as measured by the SSQ, improved in adult non-ambulatory patients with SMA types 2 and 3 after 12 months of treatment with risdiplam. A trend towards improvement was found using the bulbar subscore of the ALS-FRS-R. This is the first study to investigate bulbar function of adult patients with SMA under risdiplam treatment.

Bulbar impairment frequently occurs in adult patients with SMA but is more likely to be present in severely affected patients and in patients with an advanced disease [14, 16]. Rates of feeding and/or swallowing difficulties are reported in 30–50% of SMA type 2 and 3 patients, with rates being comparatively higher in type 2 patients [13]. Because only patients with reported swallowing dysfunction were included in this study, our sample consisted almost entirely of patients with type 2 SMA. The first available intrathecal therapy with nusinersen can be difficult to perform, especially in severely affected patients with scoliosis and spondylodesis, and constitutes relevant radiation exposure due to the need for CT-guided punctures in this patient group [25]. Consequently, in countries with access to all treatment options, a large proportion of such patients are assigned to risdiplam therapy, which is reflected in our sample’s high proportion of patients with spondylodesis. Real-world as well as pivotal study populations of adult SMA patients receiving risdiplam are, on average, in a more severe disease stage than those receiving nusinersen, reflected by a predominance of type 2 SMA and lower RULM and especially HFMSE baseline scores. This was also the case in the CHERISH and SUNFISH pivotal trials on the late-onset forms of SMA under nusinersen and risdiplam, respectively [26, 27].

HFMSE and RULM scores were lower in our sample than in comparable studies investigating adult SMA patients, such as real-world studies on nusinersen [9, 10], indicating the advanced disease stage in a real-world setting of adult SMA type 2 and 3 patients with bulbar impairment. Motor scores were also lower than those in our previous study on bulbar function of adult type 2 and 3 SMA patients treated with nusinersen, which we conducted using the same study approach [16]. While the average age of 34 years in this risdiplam cohort and 38 years in our former nusinersen cohort as well as the disease duration with an average of 32 and 38 years were very similar, the motor level with a baseline HMFSE score of 1.96 and RULM score of 8.84 in this risdiplam cohort was considerably lower than that of our nusinersen cohort with an HFMSE score of 8.57 and RULM score of 12.65. Comparable studies addressing adult patients with SMA under treatment with risdiplam are not yet available. In the pivotal SUNFISH study, which included patients with type 2 and 3 SMA, the proportion of adult patients was small and significantly younger. The baseline motor scores were also higher in that study [27]. The improvement in the RULM score that we observed after 12 months of risdiplam therapy was significant; however, with a mean difference of 0.3 points, it was small and not considered clinically meaningful [28], aligning with the low baseline motor level of the selected study cohort. Considering the above-mentioned sample characteristics, it is noteworthy that an improvement in swallowing quality could still be observed with risdiplam, despite the advanced and severe disease stage of the patients in our study. We did not find any improvement but preserved bulbar function after treatment with nusinersen in our earlier investigation using the same measures [16]. However, it is unclear to what extent a lack of improvement here can be attributed to the effectiveness of a therapy or to the patients’ limited capacity to improve. Further data on bulbar function in adult patients with SMA under gene-based therapies are scarce. Data available for nusinersen also support an overall positive effect on bulbar symptoms in adult patients with SMA, yet not as clearly and robustly [29–32]. A direct comparison of both therapies is challenging due to the significant differences in the clinical features of the two patient groups.

Reliable data on the natural history of bulbar function in adult patients with SMA that would allow a more precise interpretation of our findings are not available. SMA is a chronic progressive disease throughout adulthood, with a decline in motor scores over months or years, depending on the clinical type and age of the patients [6, 33, 34]. Bulbar dysfunction often progresses rapidly in untreated patients with type 1 SMA, who regularly switch from oral feeding to alternative nutrition during the natural disease course [14]. In milder phenotypes, bulbar symptoms often occur later during the course of the disease [35].

To assess bulbar function, we chose the SSQ and b-ALSFRS-R, which have already been used to evaluate bulbar function in adult patients with SMA treated with nusinersen [16]. The SSQ is a questionnaire to detect oropharyngeal dysphagia of various causes and allows for a standardized and patient-centered assessment of the severity of bulbar dysfunction and swallowing quality. It is easy to obtain and has been shown to be applicable for screening oropharyngeal dysphagia in several neuromuscular diseases [36]. It has also been shown to be useful in detecting dysphagia in adult patients with Duchenne muscular dystrophy and ALS [37, 38]. For SMA and other motor neuron diseases, the scores of most individual items correlated with the total SSQ score [36]. While the SSQ assesses swallowing in detail, other bulbar components, such as speech and salivation, are not directly addressed [21]. Therefore, this score does not cover the entire bulbar spectrum. Components such as the mandibular range of motion, masticatory and tongue forces, and head posture, identified as relevant bulbar function components in previous studies [15, 39–41], are only indirectly assessed. As a complement, the b-ALSFRS-R assesses speech, salivation, and swallowing using one item each. Like the SSQ, it is a patient-reported outcome measure. The separate consideration of different subscores of the ALSFRS-R enables precise assessment of different functional domains [42]. While primarily used for ALS, the ALSFRS-R has been regularly applied and validated for the clinical assessment of SMA patients [11, 43, 44]. The *SMA Functional Rating Scale* (SMA-FRS) was developed specifically for SMA but disregards bulbar function [45]. The broader measurement of bulbar function by the b-ALSFRS-R score compared to the SSQ may explain why we did not find a significant improvement in this score over the 12-month observation period. The different resolutions and variabilities of the two scores can also affect their correlation with the baseline motor scores, which were significant for the b-ALSFRS-R, but not for the SSQ. However, the correlation between the two bulbar scores supports the consistency in their evaluation of bulbar function.

Reports on pathophysiological correlates of bulbar impairment in patients with SMA comprise a variety of examination methods and underlying mechanisms ranging from mere morphological changes to altered function of the infra- and suprahyoidal as well as masticatory muscles, nasopharynx, and laryngeal closure to bolus transport and transit into the esophagus [14]. The regulation of such processes is very complex and involves areas of the brainstem and motor neurons within the trigeminal, facial, and hypoglossal motor nuclei, the nucleus ambiguus and vagus, and the cervical myelon [46]. Risdiplam might positively affect these involved components despite already advanced neuronal degeneration. Thinking about possible explanations, one advantage of risdiplam in this regard could be its systemic distribution and increased SMN protein concentration in the central nervous system as well as in peripheral tissues, including muscles [47]. This might also positively influence neuromuscular junction dysfunction, which appears to play a role in SMA pathogenesis and is often associated with bulbar symptoms, for example in myasthenia gravis [48]. However, these are purely hypothetical thoughts without our results being sufficient to prove such associations.

Some limitations should be considered when evaluating the results of this study. The study's statistical power was limited due to the sample size of 25 patients and the recruitment of patients at a single site. Nonetheless, assessment and treatment standards, including additional speech and physical therapy, were consistent and uniform. The observation period covered only 12 months for a slowly progressive disease that had already lasted for several years on average, leaving it unclear how bulbar function will develop over a longer period. However, long-term improvements in motor function have been observed following gene-based therapies in adults with SMA [49]. The SSQ has not yet been validated specifically for SMA. It was selected in addition to the b-ALSFRS-R based on the aforementioned considerations. Both measuring tools are patient-reported outcome measures and are thus limited in their objectivity and could be biased by patients' expectations of a new therapy. Furthermore, a recent study assessing oro-bulbar involvement in adults and children with SMA using objective measures such as lip and tongue strength and mouth opening revealed that patients´ perception of bulbar function often differs from objectifiable deficits, some of which are not subjectively perceived by patients [32]. This limits the interpretability of patient-reported outcome measures in this field. The potential impact of enhanced neck muscle strength and improved posture, aspects that were not specifically examined, on the quality of swallowing cannot be ruled out. However, the lack of meaningful improvement in general motor scores mitigates this assumption.

Our findings provide considerations for future research in this field. Concerning the limited objectivity of the measures of bulbar function we used, it seems that combining more objective measuring methods would be beneficial for validating the data. For this purpose, instrumental examinations, such as measuring bite force and mouth opening, videofluoroscopic examination using flexible endoscopic evaluation of swallowing (FEES), and image-based methods, including real-time MRI, might be useful [29, 50]. However, some assessments require considerable effort to conduct and evaluate. Preferably, assessments should be quick and easy for patients to perform. A recent study in a large sample revealed maximum bite force, tongue pressure, and mouth opening as objective measures that can not only discriminate between healthy individuals and SMA patients but also between different SMA types [29]. Further multicenter studies with larger sample sizes and longer observation periods are required to strengthen our conclusions. A direct comparison of the available gene-based therapies with respect to their effects on bulbar function in adults could improve our understanding of therapy and individualized management.

## Conclusion

We demonstrated an improvement in subjective swallowing quality, as measured by the SSQ, over 12 months of risdiplam therapy in SMA patients with an advanced disease stage. This suggests that risdiplam has a particularly positive effect on bulbar function. It will be of great importance in clinical practice as well as in future studies to assess bulbar function in addition to the established motor scores to reveal additional meaningful treatment effects, especially in severely affected patients, and to support more individualized therapeutic decision-making. The SSQ and b-ALSFRS-R have been shown to be suitable and easy to apply but may be supplemented by more objective assessments of bulbar function in the future.

## Data Availability

The data presented in this study are available on request from the corresponding author.
